# Beneficial Effects of Synbiotics on the Gut Microbiome in Individuals with Low Fiber Intake: Secondary Analysis of a Double-Blind, Randomized Controlled Trial

**DOI:** 10.3390/nu16132082

**Published:** 2024-06-29

**Authors:** Aakash Mantri, Linda Klümpen, Waldemar Seel, Peter Krawitz, Peter Stehle, Bernd Weber, Leonie Koban, Hilke Plassmann, Marie-Christine Simon

**Affiliations:** 1 Institute of Nutrition and Food Science, Nutrition and Microbiota, University of Bonn, 53115 Bonn, Germany; 2 Institute for Genomic Statistics and Bioinformatics, University Hospital Bonn, 53127 Bonn, Germany; 3 Institute of Nutrition and Food Science, Nutritional Physiology, University of Bonn, 53115 Bonn, Germany; 4 Institute of Experimental Epileptology and Cognition Research, University of Bonn, 53115 Bonn, Germany; 5 Center for Economics and Neuroscience, University of Bonn, 53113 Bonn, Germany; 6 Lyon Neuroscience Research Center (CRNL), Centre National de la Recherche Scientifique (CNRS), Institut National de la Santé et de la Recherche Médicale (INSERM), Université Claude Bernard Lyon 1, 69500 Lyon, France; 7 Institut Européen d‘Administration des Affaires (INSEAD), 77300 Paris, France; 8 Control-Interoception-Attention Team, Paris Brain Institute (ICM), 75013 Paris, France

**Keywords:** synbiotics, fiber, intervention study, gut microbiota, probiotic, prebiotic, dysbiosis

## Abstract

Insufficient dietary fiber intake can negatively affect the intestinal microbiome and, over time, may result in gut dysbiosis, thus potentially harming overall health. This randomized controlled trial aimed to improve the gut microbiome of individuals with low dietary fiber intake (<25 g/day) during a 7-week synbiotic intervention. The metabolically healthy male participants (*n* = 117, 32 ± 10 y, BMI 25.66 ± 3.1 kg/m^2^) were divided into two groups: one receiving a synbiotic supplement (Biotic Junior, MensSana AG, Forchtenberg, Germany) and the other a placebo, without altering their dietary habits or physical activity. These groups were further stratified by their dietary fiber intake into a low fiber group (LFG) and a high fiber group (HFG). Stool samples for microbiome analysis were collected before and after intervention. Statistical analysis was performed using linear mixed effects and partial least squares models. At baseline, the microbiomes of the LFG and HFG were partially separated. After seven weeks of intervention, the abundance of SCFA-producing microbes significantly increased in the LFG, which is known to improve gut health; however, this effect was less pronounced in the HFG. Beneficial effects on the gut microbiome in participants with low fiber intake may be achieved using synbiotics, demonstrating the importance of personalized synbiotics.

## 1. Introduction

The human gut microbiota comprises trillions of microorganisms. More than 90% of bacterial species belong to one of the four phyla: Bacillota (synonym: Firmicutes), Bacteroidota, Actinobacteria, and Pseudomonadota [1]. The microbiome encompasses all microbial genes and is linked to the onset of diverse diseases, including type 2 diabetes and non-alcoholic steatohepatitis. However, the composition of the gut microbiome can also be modulated by the intake of prebiotics, probiotics, and synbiotics [2].

Synbiotics, which combine pre- and probiotics, exert beneficial effects on host health, synergistically enhancing outcomes beyond those achieved by either probiotics or prebiotics alone [2]. Additionally, prior research indicates that synbiotics may possess therapeutic potential for conditions such as insulin resistance and inflammatory bowel syndrome [3,4,5]. However, whether synbiotics can also be used for disease prevention owing to their microbiome-modulating properties remains unknown [6,7].

The interaction between nutrition and the microbiome is increasingly being recognized as an important environmental factor for human health. In particular, the type, origin, and quality of food consumed shape the composition of the intestinal microbiota [8]. Interest is growing in using specific nutritional strategies to modulate the microbiota for improving health and preventing or treating diseases. For example, fiber is an important factor in nutrition, as fiber has positive effects on cholesterol metabolism [9]. Consistent, long-term fiber consumption has been demonstrated to influence the gut microbiota by modifying bacterial fermentation, colony size, and species composition [9,10,11,12]. Specifically, there was an increase in the abundance of bacteria that degrade polysaccharides and produce short-chain fatty acids (SCFAs), which are considered beneficial [13,14].

Furthermore, a higher intake of fiber is linked to the prevention of diseases such as type II diabetes mellitus or heart diseases and is considered beneficial [15,16]. Additionally, the evidence-based guidelines of the German Association of Nutrition (DGE) on carbohydrate intake indicate that high fiber intake has protective effects on the risk of developing hypertension, malignant tumors in the colorectum, obesity, coronary heart disease, and dyslipidemia [17,18,19]. The World Health Organization (WHO), Food and Agriculture Organization (FAO), and European Food Safety Authority (EFSA) all recommend a minimum daily dietary fiber intake of 25 g.

In contrast, low fiber intake has been independently linked to negative health consequences of a microbial response in the gut, including overall lower microbial diversity and richness [8]. Additionally, earlier studies have demonstrated that specific taxonomic changes can occur, especially in microbes responsible for SCFA production, resulting in decreased SCFA levels [14,20]. Against this background, this study investigated whether the intake of synbiotics for seven weeks increased the diversity of the gut microbiome and thus contributed to a healthier microbiome in human participants with an initially low dietary fiber intake. We further tested whether the administration of synbiotics had an additional positive effect on the microbiome despite the high-fiber diet [13]. 

## 2. Methods

### 2.1. Study Design and Intervention

Between March and November 2019, a randomized, placebo-controlled, double-blind study was conducted at the University Hospital Bonn, Germany [21]. Aim of this secondary analysis was to investigate the effect of a synbiotic on gut microbiome in participants with low fiber intake.

After the first session, participants were randomly assigned in a 1:1 ratio to either the synbiotic (SYN) group or the placebo (PLA) group. Both participants and investigators were blinded to the group assignments. The commercially available dietary synbiotic supplement Biotic Junior, provided by the manufacturer MensSana contained 2 × 10^9^ colony forming units (CFU) probiotic bacteria from five strains (*Bifidobacterium lactis*, *Lactobacillus acidophilus*, *Lactobacillus casei*, *Lactobacillus salivarius*, *Lactococcus lactis*) along with prebiotic inulin derived from agave. The placebo product was identical in appearance and taste and contained microcrystalline cellulose (MCC). The supplements were administered once daily (2 g dissolved in water) for seven weeks. The participants were instructed to maintain constant dietary behaviors and physical activities during the study phase. Anthropometric measurements, dietary records, and fecal samples were gathered from participants both before and after the seven-week intervention period. 

The study adhered to the Declaration of Helsinki guidelines and received approval from the Ethics Committee of the University Clinic Bonn (number 347/18). Before the study commenced, written informed consent was obtained from all participants. The trial was preregistered on the Open Science Framework (https://osf.io/utsn4, accessed on 10 April 2024) with a comprehensive outline of the protocol.

### 2.2. Participants

Eligible participants were males between the ages of 20 and 60, non-smokers, with a BMI ranging from 20 to 34 kg/m^2^, not following specific dietary restrictions (such as vegetarianism) or having food allergies or intolerances. Additionally, they had not taken any hormonal medication or antibiotics in the four weeks preceding the study entry. 

### 2.3. Dietary Intake

Participants were responsible for recording their dietary intake, following instructions to complete a three-day food protocol before both the initial and subsequent sessions. The data collected were transferred and preprocessed utilizing the nutritional software EBISpro 2016.

### 2.4. Fiber Group Definition

**Figure 1:**
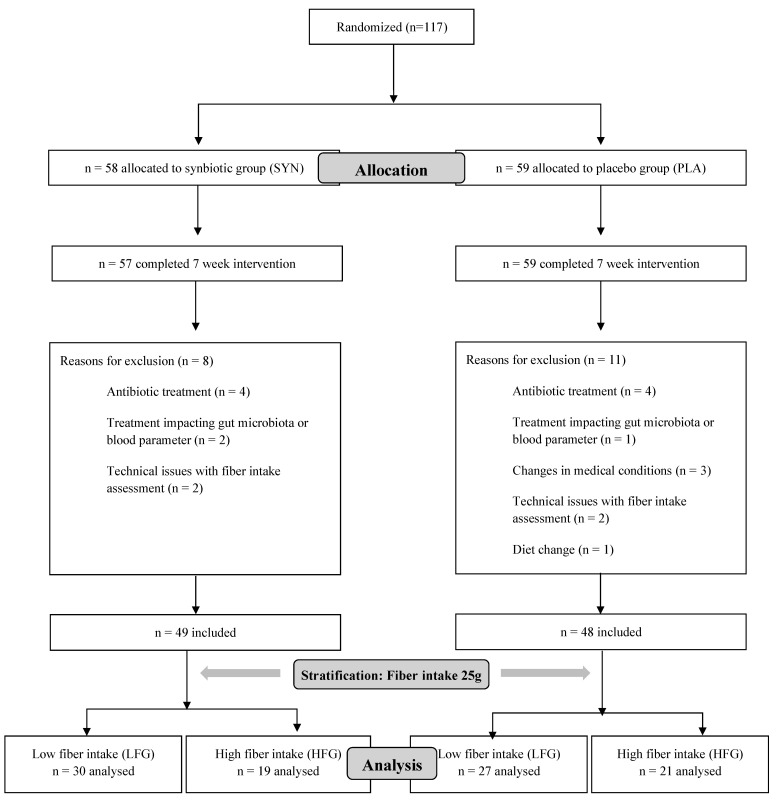
Flow chart of the study population from randomization to analysis.

For this secondary analysis, the participants were divided into a low or high fiber group depending on their initial fiber intake, based on fiber recommendations for adults, by WHO, FAO, and EFSA, with the following criteria for stratification: low fiber group (LFG): <25 g/day and high fiber group (HFG): ≥25 g/day (Figure 1).

### 2.5. Anthropometrics 

Anthropometric measurements were recorded by trained personnel according to the standardized procedures. Body height and weight were measured at 0.1 kg, respectively. BMI was computed using the formula BMI = weight [kg]/height [m]^2^. Body weight and fat percentage were assessed using a medical-grade bioimpedance scale (Tanita Europe BV, Amsterdam, The Netherlands).

### 2.6. Gut Microbiome Sample Processing

To analyze the gut microbiome, stool samples were collected within 24 h before each study visit using a standardized procedure. These samples were promptly frozen at −80 °C until analysis. DNA extraction was carried out using a QIAamp PowerFecal DNA Kit following the manufacturer’s protocol (Qiagen, Hilden, Germany) [22]. In summary, stool samples were mechanically disrupted using Bead Tubes with a 0.7 mm Dry Garnet. High-throughput sequencing of the V3V4 region of the 16S rRNA gene was performed using the primer pair 341f-806bR. QIIME2 (Quantitative Insights into Microbial Ecology; version 2023.5) [23] was utilized for all preprocessing tasks. The 300-bp paired-end reads generated from the MiSeq analysis were assembled using DADA2 [24]. DADA2 was employed for quality filtering of paired-end reads, employing a quality threshold of >30 and eliminating mismatched barcodes. The resulting Amplicon Sequence Variants (ASVs) from DADA2 were utilized for subsequent analysis. Additionally, a phylogenetic tree was constructed using these ASVs. Ultimately, the Silva taxonomy database (version 138) [25,26] was employed for taxonomic assignment of sequences across all taxonomic levels, estimating their relative abundances. 

### 2.7. Statistical Analyses

To complete the secondary analysis as discussed above, diversity metrics such as Shannon index and Faith’s phylogenetic diversity (Faith’s PD). Microbiome composition metrics such as Jaccard distance, abundance of each microbe, dietary fiber intake were considered as outcome variables.

#### 2.7.1. Analyzing Participant’s Characteristics

All statistical analyses were conducted using R Studio (version 4.2.2, Boston, MA, USA). Continuous data were presented as mean ± standard deviation (SD), while categorical variables were expressed as frequencies. Normal distribution of continuous variables was assessed using the Shapiro–Wilk test. Baseline differences between treatment groups were evaluated using an unpaired Student’s *t*-test for normally distributed variables, Mann–Whitney test for non-parametric variables, and Pearson’s chi-square test for categorical variables. To mitigate the potential impact of dietary intake variations on microbiome changes, relative changes in energy, carbohydrates, protein, and fat intake, as well as changes in body weight, BMI, and fat mass between groups, were compared using the *t*-test or Wilcoxon test. Statistical significance was set at *p* < 0.05.

#### 2.7.2. Diversity Analysis

For diversity analysis, rarefaction was conducted at a sampling depth of 12,000 sequences, leading to the exclusion of 21 samples from the analysis. Alpha diversity metrics, including the Shannon index and Faith’s PD), along with beta diversity metrics such as Jaccard distance, were calculated using the QIIME2 package. To analyze gut microbial composition and confirm the intervention’s impact on the gut microbiome in a longitudinal manner, a linear mixed model was employed to examine group differences in microbial alpha-diversity (Shannon index, Faith’s PD). Furthermore, analyses of beta-diversity (Jaccard distance) incorporated baseline synbiotic abundance and two-way interactions (group × baseline-synbiotic-abundance). The placebo group was designated as the reference, and synbiotic abundance was characterized as the total relative abundance of synbiotic bacteria in the gut microbiome before the intervention (at baseline).

#### 2.7.3. Gut Microbiome Analysis Using sPLS-DA

To investigate the effect of synbiotics on abundances of different gut microbes in each fiber group, a dimensional reduction technique, similar to principal component analysis, was performed using a sparse partial least square discriminatory analysis (sPLA-DA), implemented in the mixOmics v6.8.5 R package [27]. Using sPLS-DA is advantageous because it effectively handles the complex relationships among many microbes, reduces data complexity, and considers multiple microbes at once, providing a more robust and comprehensive analysis of the gut microbiome data. This approach also offers clear visualizations and highlights key contributors, enhancing the interpretation and validity of our findings.

In this analysis, components are formed by maximizing covariance between the microbes, to distinguish intervention groups from each other. In our case, a partial least square model with feature selection using LASSO regularization technique was performed on the high dimensional microbiome dataset. For selecting the optimal number of components and microbes in each component needed to distinguish between intervention groups, a fivefold cross-validation with 50 repeats was performed. The optimal number of components and regularization was selected minimizing the balanced error rate (BER). To check for the stability of the selected microbiomes in the previous step, a final tenfold cross validation with 50 repeats was performed. The evaluation of the model was done using the BER, as well as the AUC. Sample plots and loading plots are made using the functions plotIndiv and plotLoadings from mixomics package, respectively. More detailed workflow can be found in the mixOmics tutorial (http://mixomics.org/case-studies/splsda-srbct-case-study/, accessed on 15 March 2024). This analysis was performed at different taxonomic levels with a relative abundance threshold of 0.01%. Change in center-log-ratio transformed microbiome data between sessions (endline-baseline) was provided as an input. Participants without postintervention data were excluded (*n* = 1).

#### 2.7.4. Metabolic Pathway Analysis Using sPLS-DA

Phylogenetic Investigation of Communities by Reconstruction of Unobserved States 2 (PICRUSt2) v2.5.1 was employed to predict the functional potential of microbial communities. Additionally, Kyoto Encyclopedia of Genes and Genomes (KEGG) orthologs were identified, and KEGG pathways were inferred from these orthologs using KEGG Orthology (KO), a classification system developed based on the KEGG database [28]. The ko2kegg_abundance function in the ggpicrust2 package [29] was used for this task. sPLSA-DA was used for these KEGG pathways.

#### 2.7.5. Individual Microbe Analysis Using NBZIMM

Compared to a system level analysis such as sPLS-DA, we additionally performed a regression analysis at feature level. At feature level, each gut microbe was considered as an independent microbe and the regression analysis was performed on each individually. Regression analysis of gut bacterial taxa was conducted using a negative binomial and zero-inflated mixed model (NBZIMM) [30] involving the gut microbiomes of 97 participants. Taxa were deemed adequately abundant if each taxon was present in at least 20% of the samples [10]. This model addresses zero-inflation concerns for certain microbiome taxa and consists of two steps: first, a logistic model predicts excess zeros, followed by a negative binomial distribution for overdispersed counts. The model, incorporating the effects of group, session, and group × session, was utilized to pinpoint microbial taxa significantly impacted by the intervention. Age and BMI served as covariates, and adjustments were made for varying sequence counts in each sample. Additionally, random effects were included to account for repeated sampling of the microbiota from the same individual. The model was applied individually to each taxon, and to counteract multiple testing, a false discovery rate (FDR)-adjusted *p* < 0.05 was chosen for the associated genus.

## 3. Results

### 3.1. Participant’s Characteristics

Out of the initial 117 participants, 16 were excluded for various reasons: antibiotic treatment during the intervention (*n* = 8), changes in medical conditions or treatment affecting the gut microbiota (*n* = 6), reported changes in dietary habits (*n* = 1), or non-attendance at the post-intervention session (*n* = 1). Additionally, four individuals were excluded due to technical issues with fiber intake assessment, resulting in a final analysis of 97 participants, with an average age of 32 ± 10.69 years (Table 1). No participants reported any adverse effects after taking synbiotics or the placebo. At baseline, there were no significant differences between the synbiotic and placebo groups within each fiber group regarding anthropometric data including weight, BMI, and body fat mass. There was no significant difference in microbial diversity between the intervention groups at baseline within each fiber group. 

At baseline, the HFG and LFG were observed to have partially separated microbiomes at the genus level (Figure S1A). Among the top five genera contributing to the components driving the separation, *Butyrivibrio*, *Lachnospiraceae UCG-008*, and *Lachnospiraceae UCG-008* were found to have a higher mean abundance in HFG and *Dorea* and *Negativibacillus* in the LFG group (Figure S1B). Similar separation of the two groups at baseline was also observed at the species and ASV levels (Figure S1C–F). At baseline, there were no differences between HFG and LFG in macro-nutrients intake, apart for carbohydrates (LFG: 279.89 ± 85.51 g, HFG: 323.95 ± 68.22 g, t = −11.00, *p* = 1.92 × 10^−15^) and fiber (LFG: 19.54 ± 3.66 g, HFG: 32.74 ± 6.94 g, t = −2.80, *p* = 2.72 × 10^−3^), which was expected, as we had stratified accordingly. However, protein (LFG: 114.12 ± 44.29 g, HFG: 111.15 ± 25.86 g, t = 0.41, *p* = 0.68), fat (LFG: 115.99 ± 30.75 g, HFG: 126.4 ± 34.28 g, t = 78.36, *p* = 0.12), and alcohol intake (LFG: 11.57 ± 19.06 g, HFG: 5.82 ± 9.96 g, t = 88.75, *p* = 0.56) were similar between HFG and LFG.

### 3.2. Anthropometric Measures and Macro-Nutrient Intake

Since the participants were instructed to maintain their usual isocaloric diet during the 7-week intervention period, there were no differences in the relative changes in body weight, BMI, or fat mass between the fiber groups. Similarly, no significant differences in the relative changes in energy, carbohydrate, protein, and fat intakes were observed in either fiber group (Table 2).

### 3.3. Results of the Diversity Analysis

As expected, the baseline composition of the gut microbiome exhibited individual variation (Shannon index: 3.81 min, 7.88 max). However, it was similar between the intervention groups (Jaccard distance: 0.89 ± 0.02 sd) for both the LFG and HFG groups. Upon intervention, the gut microbiome composition was altered in the LFG (Figure S2A), whereas the microbiome of the HFG remained unaffected by the intervention (Figure S2B). This alteration in LFG was depended on baseline synbiotic abundance. 

### 3.4. Synbiotic-Induced Changes in Gut Microbiome

To analyze the changes in the gut microbiome composition from session one to session two at the genus, species, and ASV levels, sPLS-DA was applied on the gut microbiome. Results from these models are shown in sample plots and loading plots. Sample plots show how the changes in the microbiome separate the groups. The loading plots highlight the microbes whose changes were most influential in differentiating between the two groups. 

#### 3.4.1. Microbiome Changes in LFG

**Figure 2:**
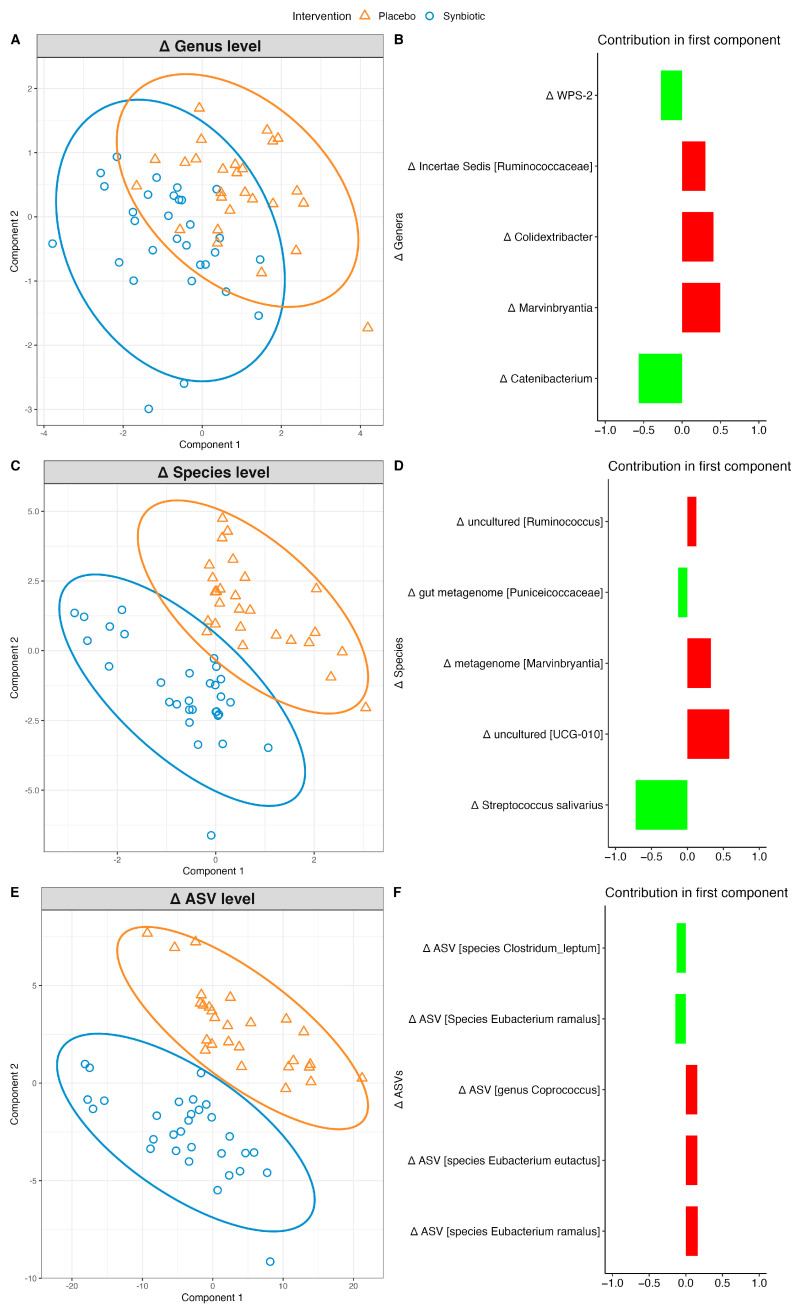
Intervention effect on gut microbiome in LFG at genus, species, and ASV levels. Sample plots with 95% confidence ellipses for each taxonomy level are displayed at (**A**,**C**,**E**), showing the changes in gut microbiome that separates the intervention groups (synbiotic and placebo are displayed as blue and orange respectively), and their corresponding loading plots at (**B**,**D**,**F**) derived from sPLS-DA models. The loading plot displays which changes in bacterial abundance are most important in differentiating the intervention groups. The top 5 contributors are displayed in the loading plot. Green/red displays mean increase/decrease in abundance of the microbe in synbiotic relative to placebo, respectively.

A microbiome signature that separated the intervention groups was observed at the genus (AUC: 0.63, *p*-value: 0.07), species (AUC: 0.68, *p*-value: 0.01), and ASV levels (AUC: 0.67, *p*-value: 0.02). The separation between the synbiotic and placebo groups was the most pronounced at the ASV level (Figure 2A–F, Table S1).

At the ASV level, the greatest decrease was observed in the species *Clostridium leptum* (genus *Incertae sedis*) in the synbiotic group compared to the placebo group (Figure 2B, Table S2). This trend was also observed at the species and genus levels (Figure 2C,E). A decrease in three ASVs and an increase in one ASV belonging to the genus *Faecalibacterium* was observed in synbiotic group compared to those in the placebo group. Furthermore, alterations were observed in ASVs belonging to *Eubacterium ramulus* in the synbiotic group compared with the placebo group. 

At the species level, an increase was observed in *Streptococcus salivarius* in the synbiotic group compared to the placebo group, whereas a decrease was observed in *UCG-010*, *Ruminococcus*, *Marvinbryantia* in the synbiotic group compared in the placebo group (Figure 2D). Among the top five genera contributing to the components, an increase was observed in the abundance of *WPS-2*, *Alloprevotella*, and *Catenibacterium*, whereas a decrease was observed in the abundance of *Marvinbryantia*, *Colidextribacter*, and *Ruminococcaceae incertae sedis* in the synbiotic group compared to those in the placebo group. 

#### 3.4.2. Microbiome Changes in HFG

**Figure 3:**
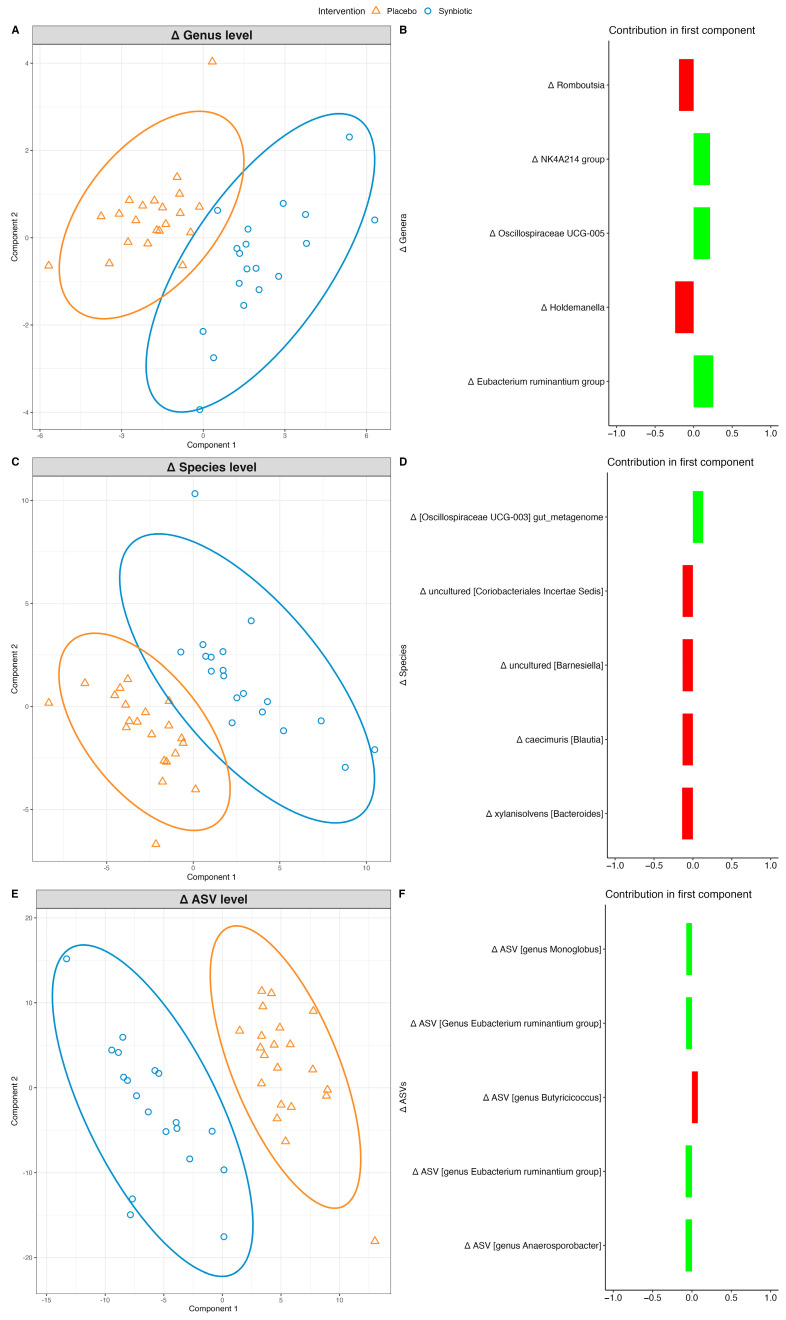
Intervention effect on gut microbiome in HFG at genus, species, and ASV levels. Sample plots with 95% confidence ellipses for each taxonomy level are displayed at (**A**,**C**,**E**), showing the changes in gut microbiome that separates the groups (synbiotic and placebo are displayed as blue and orange respectively), and their corresponding loading plots at (**B**,**D**,**F**) derived from sPLS-DA models. The loading plot displays which changes in bacterial abundance are most important in differentiating the groups. The top 5 contributors are displayed in the loading plot. Green/red displays mean increase/decrease in abundance of the microbe in synbiotic relative to placebo, respectively.

Similar to LFG, microbiome signature separation was observed at the genus (AUC: 0.78, *p*-value: 2.0 × 10^−3^), species (AUC: 0.80, *p*-value: 1.3 × 10^−3^), and ASV levels (AUC: 0.68, *p*-value: 0.04) following intervention. Again, a well-defined microbiome separation between the synbiotic and placebo groups was observed at the ASV level (Figure 3A–F).

Synbiotic intervention affected multiple ASVs, including an increase in two ASVs from the genus *Eubacterium ruminantium* in the synbiotic group compared to that in the placebo group. This increase in abundance was also observed at the species and genus levels. A decrease in ASV from the genus *Holdemanella* was observed in the synbiotic group compared to that in the placebo group, which was also observed at the genus level. Another decrease in uncultured species from the *Rikenellaceae RC9* gut group was detected in the synbiotic group compared to that in the placebo group. A decrease in the genus *Bacteroides* as well as in the two species *Bacteroides xylanisolvens* and *Bacteroides vulgatus* was observed in the synbiotic group compared to the placebo group. 

### 3.5. Synbiotic-Induced Changes in Microbiome-Derived Metabolic Pathways

**Figure 4:**
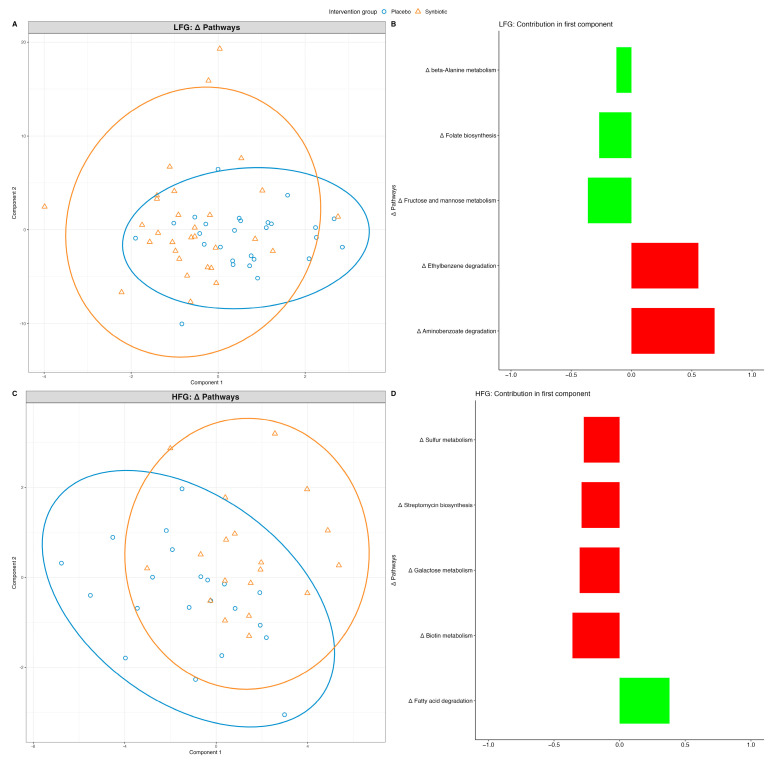
Intervention effect on gut microbiome at the pathway level in LFG and HFG. Sample plots with 95% confidence ellipses for each taxonomy level are displayed at (**A**,**C**), showing the changes in gut microbiome that separates the groups (synbiotic and placebo are displayed as blue and orange respectively), and their corresponding loading plots at (**B**,**D**) derived from sPLS-DA models. The loading plot displays which changes in bacterial abundance are most important in differentiating the groups. The top 5 contributors are displayed in the loading plot. Green/red displays mean increase/decrease in abundance of the microbe in synbiotic relative to placebo, respectively.

Only slight intervention effects were observed in the predicted KEGG pathways in the synbiotic and placebo groups within the HFG and LFG groups (Figure 4A,C). There was an increase in fructose and mannose metabolism, whereas aminobenzoate degradation decreased in the synbiotic group compared to that in the placebo in LFG (Figure 4B). Similarly, in the HFG, only the fatty acid degradation increased, whereas biotin metabolism, galactose metabolism, streptomycin biosynthesis, and sulfur metabolism decreased in the synbiotic group compared to the placebo group (Figure 4D).

### 3.6. Individual Alterations for Gut Microbiome

Taxonomic analysis at the genus level revealed that the most abundant genera, the core microbiome (top 11 microbes), remained stable in the LFG and HFG (Figure S2D,E). In the LFG, this intervention led to a significant increase in the abundance of seven genera and a decrease in the abundance of six genera. Similar to the LFG, the intervention effect on the HFG increased the abundance of nine genera and significantly decreased the abundance of 14 genera in the HFG (Table S3).

## 4. Discussion

In this secondary analysis of this randomized, placebo-controlled, double-blind intervention study, it was observed that synbiotics may exert beneficial effects on gut microbiome in participants with low fiber intake, depending on the baseline microbiome, indicating the need for personalized synbiotics. Thus, after 7 weeks of intervention, there was a significant increase in the abundance of SCFA-producing microbes in the LFG, which improve gut health; however, this effect was less pronounced in the HFG. Accordingly, these results suggest that the increase in the abundance of beneficial microbes such as *Catenibacterium* and *Alloprevotella* induced by synbiotics may counteract the low initial microbial diversity as a result of low fiber intake. This is in line with other dietary intervention studies showing that microbes such as *Catenibacterium* and *Alloprevotella* are more abundant with high fiber intake [31,32].

Dietary fibers are crucial for maintaining human gut health. Numerous studies have explored the impact of dietary fiber on the gut microbiome and metabolic health in both mice and humans [33,34,35]. Many of these studies have demonstrated the beneficial effects of dietary fiber on health [16], on the other hand a deficiency of fiber intake may also have negative effects [36]. Low dietary fiber intake results in decreased microbial diversity [8,36,37] and different microbiome compositions compared with high dietary fiber intake, which is associated with increased microbial diversity [22,38,39]. This is in line with our results, since in our intervention study population, a partially different microbiome signature between the LFG and the HFG at baseline was also observed, which supports the hypothesis that fiber intake, per se, modulates microbial composition in humans. 

Diversity analysis showed that the success of the intervention in the LFG was highly dependent on the abundance of synbiotic strains at baseline. This indicates that the impact of synbiotics on the microbiome might be personalized depending on the person’s daily fiber intake and baseline microbiome. This is in line with the results of probiotics trials, showing a greater benefit of probiotics if particular microbes are already present at baseline [40,41,42]. 

In both LFG and HFG, a clear separation between the synbiotic and placebo groups was observed at amplicon sequence variant levels, while the core microbiome remained stable. This suggests that synbiotics affect specific microbes within an entire ecosystem. Notably, this intervention study was performed in metabolically healthy participants without any gastrointestinal symptoms or microbial dysbiosis; thus, strong microbial shifts were unlikely. 

However, in the HFG, synbiotic intervention led to a decrease in the abundance of *Erysipelatoclostridium*, which is linked to diet-induced obesity, and most studies have shown that depleting *Erysipelatoclostridium* is beneficial [43]. The synbiotic also increased the abundance of *Alistipes* and *Parasutterella*, which is in line with other dietary fiber interventions [44,45,46] and supports the potential role of *Parasutterella* in maintaining bile acid levels and regulating cholesterol metabolism. Furthermore, higher levels of *Parasutterella* in the gut microbiome correlate with improved low-density lipoprotein levels in healthy adults, ref. [47] indicating the potential metabolic benefits of synbiotics in the microbiome. 

Additionally, synbiotics also increased the abundance of *Alistipes* in the HFG, which might be beneficial because they may offer protection against conditions like fibrosis, colitis, cancer treated with immunotherapy, and cardiovascular disease [48]. The increase in the abundance of *Alloprevotella* in HFG might also be beneficial, as it is a widely found bacterium in the gut [32] and is associated with an improved intestinal barrier [49]. In line with this, a decrease in several opportunistic pathogens, such as *Erysipelatoclostridium* [43,50] and microbes associated with Crohn’s disease, such as *Ruminococcus gnavus* [51,52] has been detected, highlighting the potential synbiotic effect against diseases. 

Another significant increase was observed in *Prevotellaceae UCG-003* which has the potential to modulate intestinal inflammation [14,53,54]. Additionally, another species of *Streptococcus salivarius*, also known for its anti-inflammatory properties [55], was increased in the LFG. Furthermore, our synbiotic intervention caused the depletion of harmful bacteria, such as *Colidextribacter*, which is positively correlated with inflammatory metabolites [56]. 

Similar to dietary fiber intervention, prebiotics or probiotics, our findings of the synbiotic intervention within the LFG showed a strong increase in fiber-degrading genera, such as *Catenibacterium*, *Prevotellaceae UCG-003*, *Ruminococcaceae CAG-352*, and *Alloprevotella*, which produce SCFAs such as butyrate, thereby improving gut health and nutrient utilization [57,58,59]. In addition, in the HFG, synbiotic intervention increased the number of SCFA genera such as *the Eubacterium ruminantium group* [45]. Furthermore, the abundance of genes encoding the biosynthesis of the vancomycin group antibiotic pathway decreased after synbiotic intervention, which might be relevant. As vancomycin restricts the growth of Gram-positive bacteria, including some butyrate-producing bacteria, it leads to higher SCFA concentrations [60]. Additionally, our data indicated an increase in the abundance of multiple genera belonging to the Oscillospiraceae family, which has also been associated with multiple health benefits and SCFA production [61,62]. Furthermore, several ASVs of genus *Faecalibacterium alterations* were detected, which are commensal bacteria that produce butyrate and other SCFAs [63] in the LFG. The abundance of several strains belonging to *Eubacterium ramulus* were also altered in the LFG [64,65,66]. 

Functional analyses of the microbiome revealed several alterations in different pathways. Thus, a strong increase in the abundance of genes encoding the fructose and mannose metabolism pathways was observed in the LFG, which was expected because our synbiotic intervention contained inulin, which is a polyfructose. Therefore, inulin degradation may have increased the abundance of this pathway. In line, *Catenibacterium*, which are associated with inulin intake, were also shown to increase [67]. Furthermore, an increase in the abundance of genes encoding folate biosynthesis in LFG [68,69] might be highly beneficial because folate, as the natural form of vitamin B9, is recommended in diet to promote good health [70,71].

This study possesses several strengths. First, this nutritional intervention study was conducted according to the gold standard method for a randomized, placebo-controlled, double-blind study (RCT). Dietary fiber intake remained stable during the intervention as participants adhered to their habitual diet. Moreover, the RCT included a relatively large sample size of 97 participants in the analyses, encompassing a diverse range of BMI values, potentially representing the broader population. Lastly, the synbiotic utilized met all safety criteria, ensuring a low risk of adverse effects from the outset of the study. This was further affirmed by the absence of any reported side effects or adverse reactions by participants.

This study utilized a per protocol analysis due to the loss of 20 out of 117 subjects (approximately 17%) to follow-up. While intention-to-treat (ITT) analysis is generally preferred to preserve randomization and minimize bias, our findings still provide valuable insights for those adhering to the protocol. Additionally, as this is a secondary analysis, we focused on exploring additional insights without a formal sample size calculation, embracing the opportunity to generate new hypotheses and enhance our understanding of the data. A limitation of the study is that the duration of seven weeks only represents a mid-term intervention period and does not reflect long-term effects on the microbiome, such as adaptations, and we did not conduct a follow-up. Even though a one-year follow-up study was planned, due to the COVID-19 pandemic, only a few people attended. In addition, the collected data were not usable because of many confounding factors, such as COVID-19 vaccination and infection. Thus, studies specifically designed to investigate long-term effects are required to observe the beneficial effects of changes in the microbiome on metabolism, particularly in metabolically healthy participants. As we showed that synbiotics have a greater impact on the microbiome when already present at the baseline, the need for a personalized approach is suggested. 

## 5. Conclusions

In summary, by modulating the gut microbiome using synbiotics in an RCT, we were able to improve the gut microbiome of participants with low fiber intake, and this impact was individually dependent on baseline dietary fiber intake and the microbiome. 

## Figures and Tables

**Table 1:** Baseline characteristics of the study population.

	Total (*n* = 97)	LFG			HFG		
		SYN [*n* = 30]	PLA [*n* = 27]	*p* Value	SYN [*n* = 19]	PLA [*n* = 21]	*p* Value
Age (years)	32.27 ± 10.69	31.69 ± 10.98	34.72 ± 11.57	0.31	31.14 ± 8.87	30.48 ± 10.82	0.83
Weight (kg)	84.08 ± 11.66	85.73 ± 12.96	85.56 ± 12.76	0.96	82.8 ± 9.57	80.78 ± 9.85	0.51
BMI (kg/m^2^)	25.66 ± 3.1	25.7 ± 3.42	26.53 ± 3.06	0.34	25.28 ± 2.68	24.64 ± 2.97	0.48
Fat mass (%)	19.57 ± 5.19	19.86 ± 5.39	21.09 ± 5.11	0.92	19.32 ± 4.35	17.28 ± 5.41	0.20
Energy intake (kcal/day)	2819.56 ± 588.5	2792.05 ± 632.52	2650.43 ± 595.27	0.39	2746.63 ± 527.37	3134.24 ± 473.66	0.01
Carbohydrate intake (grams/day)	298.25 ± 81.37	283.89 ± 97.88	275.27 ± 70.21	0.70	309.09 ± 71.91	337.4 ± 63.42	0.20
Protein intake (grams/day)	112.88 ± 37.58	119.77 ± 53.07	107.59 ± 31.07	0.29	102.01 ± 21.95	119.43 ± 26.82	0.03
Fat intake (grams/day)	120.32 ± 32.5	116.7 ± 23.8	115.16 ± 37.71	0.86	115.83 ± 26.24	135.96 ± 38.33	0.06
Fiber intake (grams/day)	24.98 ± 8.37	19.6 ± 3.79	19.48 ± 3.58	0.90	32.17 ± 5.94	33.25 ± 7.84	0.63
Shannon index	6.55 ± 0.64	6.53 ± 0.62	6.66 ± 0.54	0.84	6.45 ± 0.88	6.53 ± 0.52	0.94
Faith’s PD	35.46 ± 8.67	37.71 ± 8.79	33.76 ± 8.15	0.20	34.58 ± 9.48	35.27 ± 80	0.68

BMI: Body Mass Index; LFG: Low Fiber Group; HFG: High Fiber Group; SYN: Synbiotic; PLA: Placebo; For Shannon index and Faith’s PD, a linear model adjusting for age and BMI was used, remaining metrics were measured using *t*-test. *p* value shown is unadjusted.

**Table 2:** Relative change in anthropometric measures and macro-nutrient intake.

	LFG			HFG		
	SYN	PLA	*p* Value	SYN	PLA	*p* Value
	(*n* = 30)	(*n* = 27)		(*n* = 19)	(*n* = 21)	
Weight (%)	0.15 ± 1.58	0.1 ± 2.04	0.92	0.66 ± 2.11	−0.47 ± 1.77	0.07
BMI (%)	0.15 ± 1.59	0.11 ± 2.04	0.92	0.66 ± 2.11	−0.47 ± 1.77	0.07
Fat mass (%)	−2.46 ± 10.16	−3.26 ± 7.87	0.75	−0.53 ± 6.77	−2.19 ± 8.19	0.49
Energy intake (%)	−0.24 ± 31.78	−6.62 ± 27.66	0.43	−14.46 ± 16.74	−5.63 ± 24.62	0.20
Carbohydrate intake (%)	−2.98 ± 29.93	−1.5 ± 36.57	0.86	−15.44 ± 17.99	−5.54 ± 25.24	0.17
Protein intake (%)	−3.36 ± 36.08	−5.97 ± 35.5	0.78	−15.53 ± 23.23	−3.89 ± 26.68	0.15
Fat intake (%)	1.49 ± 36.88	−7.81 ± 29.89	0.31	−12.69 ± 26.91	−8.68 ± 35.4	0.69
Fiber intake (%)	−4.19 ± 29.27	8.7 ± 53.14	0.25	0.66 ± 2.11	−0.47 ± 1.77	0.07

Mean relative change ± SD of weight, BMI, fat mass and energy and macronutrient intake from session 1 to session 2; BMI: Body Mass Index; *p* value shown is unadjusted and measured using *t*-test.

## Data Availability

The study was preregistered at Open Science Framework (OSF; https://osf.io/utsn4, accessed on 10 April 2024). Sharing of the data described in the manuscript, codebook, and analytical code is subject to official request due to data protection regulations.

## Supplementary Materials

The following supporting information can be downloaded from https://www.mdpi.com/article/10.3390/nu16132082/s1; Figure S1: Comparison of the baseline gut microbiome at the genus, species, and ASV levels. Sample plots with 95% confidence ellipses for each taxonomic level are displayed in (A,C,E), and their corresponding loading plots in (B,D,F) derived from sPLS-DA models. The loading plot displays the contribution (loading weight) of each feature selected from the first component with increasing importance from bottom to top. The top five contributors are presented in loading plots. Microbes with higher mean abundance in HFG/LFG are displayed as dark yellow/grey respectively, Figure S2: Box-plots displaying change in alpha diversity in each group in (A) LFG and (B) HFG. Estimate plots from analyses of change in gut microbiome composition in (C) LFG and (D) HFG. * Statistical significance. Data were analyzed using a linear mixed model. Relative taxonomic abundance at the genus level (prevalance:0.60, detection threshold:0.01%) in each group/time-point in (E) LFG, (F) HFG, Table S1: Characteristics and results of sPLS-DA models. Table S2: Component details of sPLS model, Table S3: Results of single feature taxonomy analysis using NBZIMM at the genus level in LFG and HFG displaying the estimate.

## Abbreviations

SCFA: short-chain fatty acid; PLA: Placebo; BMI: Body mass index; LFG: Low fiber group; HFG: High fiber group; sPLS-DA, sparse partial least square differential discriminant analysis; CLR: Center log ratio; BER: Balanced error rate; LASSO, least absolute shrinkage and selection operator; AUC: Area under the curve; PICRUSt2: Phylogenetic Investigation of Communities by Reconstruction of Unobserved States; KEGG: Kyoto Encyclopedia of Genes and Genomes; KO: Kegg Orthology; SD, standard deviation; ASV, amplicon sequence variant.
