# Supplementary figures and images for: Beneficial Effects of Synbiotics on the Gut Microbiome in Individuals with Low Fiber Intake: Secondary Analysis of a Double-Blind, Randomized Controlled Trial
Source: Nutrients. 2024 Jun 29;16(13):2082. doi: 10.3390/nu16132082 (PMC11243043; doi:10.3390/nu16132082)

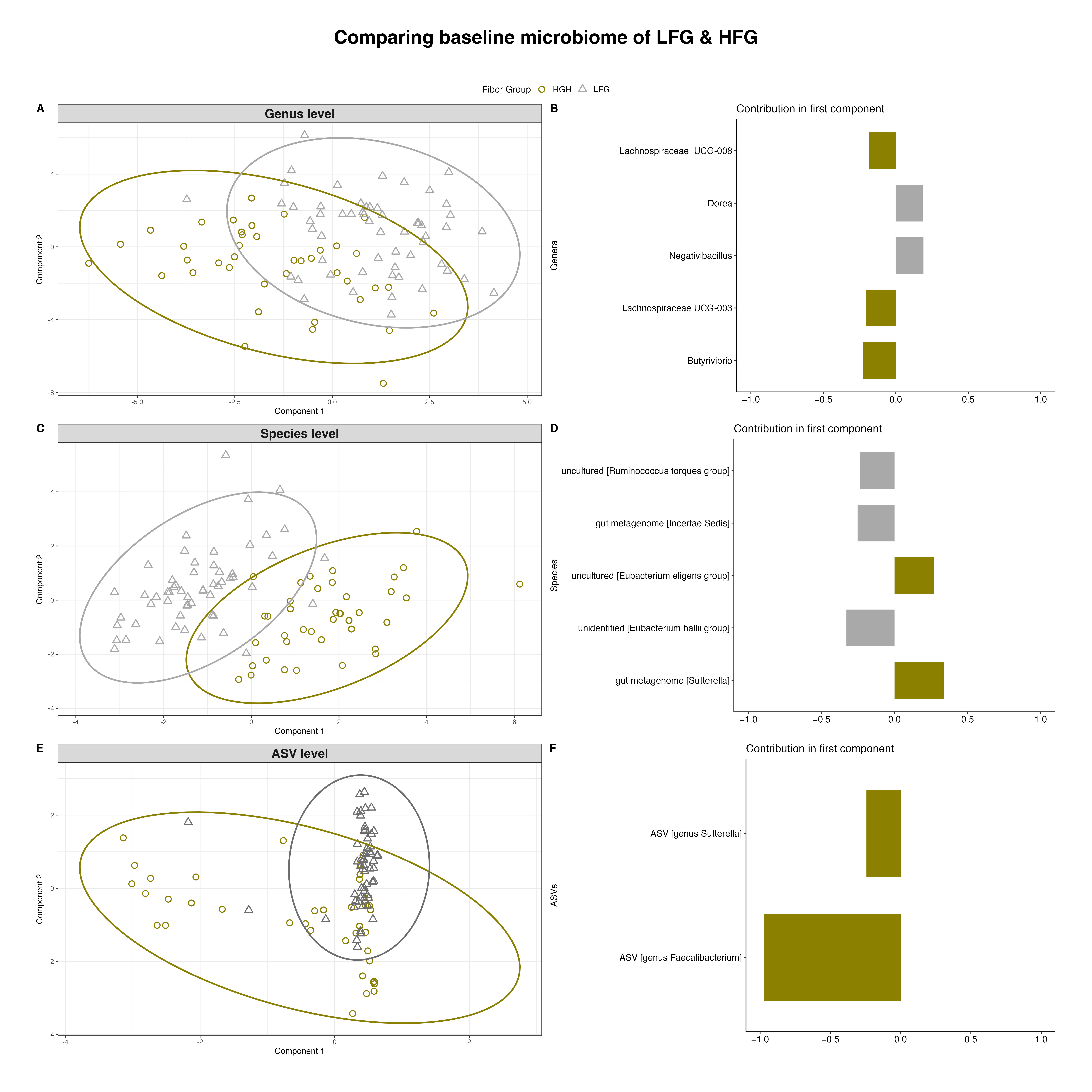

Supplement: Supplementary file 1 [file nutrients-16-02082-s001.zip › Figure S1.jpeg]

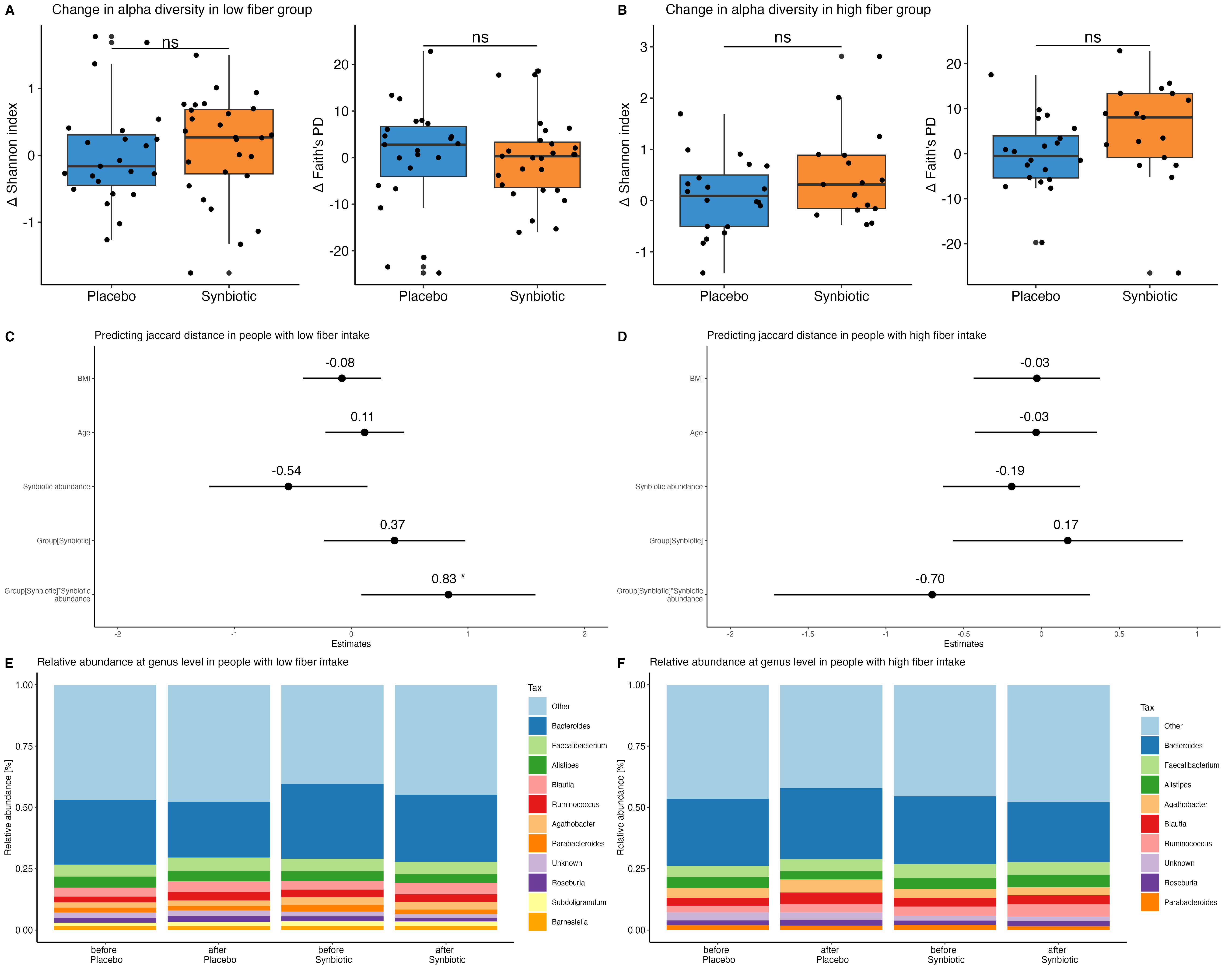

Supplement: Supplementary file 1 [file nutrients-16-02082-s001.zip › Figure S2.jpeg]
